# Quick on Your Feet: Modifying the Star Excursion Balance Test with a Cognitive Motor Response Time Task

**DOI:** 10.3390/ijerph20021204

**Published:** 2023-01-10

**Authors:** Russell K. Lowell, Nathan O. Conner, Hunter Derby, Christopher M. Hill, Zachary M. Gillen, Reuben Burch, Adam C. Knight, Jennifer C. Reneker, Harish Chander

**Affiliations:** 1 Resistance Exercise Performance Laboratory, Department of Kinesiology, Mississippi State University, Mississippi State, MS 39762, USA; 2 Neuromechanics Laboratory, Department of Kinesiology, Mississippi State University, Mississippi State, MS 39762, USA; 3 Department of Kinesiology and Physical Education, Northern Illinois University, DeKalb, IL 60115, USA; 4 Department of Industrial Systems and Engineering, Mississippi State University, Mississippi State, MS 39762, USA; 5 Human Factors and Athlete Engineering, Center for Advanced Vehicular Systems (CAVS), Mississippi State University, Starkville, MS 39759, USA; 6 Department of Population Health Sciences, John D. Bower School of Population Health, University of Mississippi Medical Center, Jackson, MS 39216, USA

**Keywords:** star excursion balance test, response times, cognitive-motor task, balance assessment

## Abstract

The Star Excursion Balance Test (SEBT) is a common assessment used across clinical and research settings to test dynamic standing balance. The primary measure of this test is maximal reaching distance performed by the non-stance limb. Response time (RT) is a critical cognitive component of dynamic balance control and the faster the RT, the better the postural control and recovery from a postural perturbation. However, the measure of RT has not been done in conjunction with SEBT, especially with musculoskeletal fatigue. The purpose of this study is to examine RT during a SEBT, creating a modified SEBT (mSEBT), with a secondary goal to examine the effects of muscular fatigue on RT during SEBT. Sixteen healthy young male and female adults [age: 20 ± 1 years; height: 169.48 ± 8.2 cm; weight: 67.93 ± 12.7 kg] performed the mSEBT in five directions for three trials, after which the same was repeated with a response time task using Blazepod™ with a random stimulus. Participants then performed a low-intensity musculoskeletal fatigue task and completed the above measures again. A 2 × 2 × 3 repeated measures ANOVA was performed to test for differences in mean response time across trials, fatigue states, and leg reach as within-subjects factors. All statistical analyses were conducted in JASP at an alpha level of 0.05. RT was significantly faster over the course of testing regardless of reach leg or fatigue state (*p* = 0.023). Trial 3 demonstrated significantly lower RT compared to Trial 1 (*p* = 0.021). No significant differences were found between fatigue states or leg reach. These results indicate that response times during the mSEBT with RT is a learned skill that can improve over time. Future research should include an extended familiarization period to remove learning effects and a greater fatigue state to test for differences in RT during the mSEBT.

## 1. Introduction

Dynamic balance is often defined as the ability to perform a task while maintaining a stable position [1,2,3]. In other words, dynamic balance is the neuromuscular response to a change in the center of gravity [4]. A widely utilized tool to evaluate deficits in dynamic balance is the Star Excursion Balance Test (SEBT). The main objective of the traditional SEBT is to maintain unilateral support while executing maximal reach distance in eight directions (anterior, medial, lateral, posterior, anteromedial, anterolateral, posterolateral, and posteromedial) with the non-supported limb. These reaching tasks are intended to challenge dynamic balance control, strength, range of motion, and proprioceptive abilities [5]. Previous studies have shown this test to be reliable to quantify lower limb functional performance [4,6,7]. The SEBT has also been used as a method of diagnoses in multiple clinical distinctions, including detecting injured versus healthy participants, to differentiate influences on performance, demonstrating outcomes from planned interventions, predicting risk of injury, and for decisions on return to play [8]. The SEBT has also been used in several research studies as the primary assessment tool for dynamic balance ability amongst athletes [1,9,10,11,12].

Different balance tests assess the integrity of different sensory and motor components of the postural control system, proper functioning of which are critical not only for athletic activities, but also for activities of daily living (ADL). ADL refer to activities practiced by individuals during their daily routine, which are important for independence and efficiency [13] and include both motor and cognitive components [14]. While the automaticity of gait was once thought to be a cognitively unattended process, recent research on those with frontal lobe damage has seen a decline in postural control and gait measures [15]. This indicates that a certain amount of higher cortical processing is required for postural control in unperturbed and perturbed stances and in ambulation. The motor performance demands, in conjunction with the cognitive processing demands, are considered dual tasking. Dual tasking has been described throughout the literature as the performance of two activities simultaneously [16]. Dual tasking interference refers to the processing interference that these single tasks have on one another, which results in detriments in performance of one or both tasks being performed [17]. In general, tasks can be divided into two categories: cognitive activities, motor activities, or an interaction of both. This interaction is known as cognitive motor interference [17].

Decrements in balance have been seen in dual tasking conditions compared to baseline or single-task conditions [18,19]. It has been suggested that a way to mitigate these detriments in dual tasking conditions is to practice them concurrently. Indeed, previous studies have examined significant improvements in dynamic balance under dual training conditions versus single-task training [13,20,21,22]. Previous studies in evaluating dual-task training in a cognitive-motor, dual-task group vs. cognitive group vs. motor group revealed significant improvements in the specific task practiced for each cognitive and motor single-task groups, but only cognitive motor dual tasking improved both cognitive and motor tasks significantly [13]. Attentional focus has also been shown to affect postural control during dual task conditions. In a recent dual task experiment, participants were instructed to focus on either the balance task or the cognitive task and displayed improved measures of postural control when attention was focused on the cognitive task as opposed to the balance task. This indicates that focusing one’s attention during a dual task scenario may be beneficial to postural control ability [23].

Given the cognitive element involved in ADLs, and also in athletic or sporting activities, balance assessment should be performed with some sort of mental/cognitive processing to interfere with the motor task, thus making the assessment a cognitive-motor balance assessment. The cognitive task chosen to interfere with a dynamic balance task should be related to what the assessment itself is trying to evaluate, which is the ability to resist falling. Response time (RT) is a critical component of dynamic balance control [2], and quantification of RT can be extremely beneficial to assess such a cognitive-motor balance task [24]. Adding a volitional cognitive response time task to the SEBT would allow for dual task assessment with an appropriate cognitive interference.

Additionally, balance-related RT and the ability to maintain dynamic balance is compromised by muscular fatigue [3,4], but their interactions are not extensively studied. Muscular fatigue has been shown to inhibit dynamic balance control during the SEBT following an isokinetic and lunging fatigue task [25]. Muscular fatigue can inhibit both somatosensory and motor responses during balance maintenance, and subsequently RT during such tasks. Impairment or slowed proprioceptive and kinesthetic sensation with fatigue can lead to slowed propagation of efferent/motor responses to help maintain balance [26]. Nonetheless, the combination dual task with response time and the SEBT as the mSEBT, along with a fatigue component, have not been simultaneously examined. Therefore, the primary purpose of this study is to modify the SEBT (mSEBT) with supplementation of a volitional, cognitive, response time task to interfere with the motor task therein, as a measure of dynamic postural control and dual task learning ability. The purpose of this study is to examine RT during a mSEBT, with a secondary goal to examine the effects of muscular fatigue on RT during the mSEBT.

## 2. Materials and Methods

### 2.1. Participants

A total of 16 healthy young male and female adults (age: 20 ± 1 years; height: 169.48 ± 8.2 cm; weight: 67.93 ± 12.7 kg; males: 5 and females: 11) participated in this study. Participants were free of any existing musculoskeletal disorders, including chronic lower extremity problems or post-surgical complications, visual, vestibular, neurological, cardiopulmonary disorders, or any hearing limitations. Any participants with these conditions were excluded. With the current study being a novel modification of the traditional SEBT, which is commonly used in athletic populations, young, recreationally trained, and otherwise healthy participants were recruited for this initial study. Sample size was determined based on prior similar balance and postural stability studies [5,27]. Prior to any data collection, all participants signed and approved an informed consent form and completed the Physical Activity Readiness Questionnaire (PAR-Q). All participants read and signed the informed consent form, approved by the Mississippi State University’s (Mississippi, MS, USA) Institutional Review Board (IRB) under the protocol IRB# 21-320.

### 2.2. Procedures

**Figure 1:**
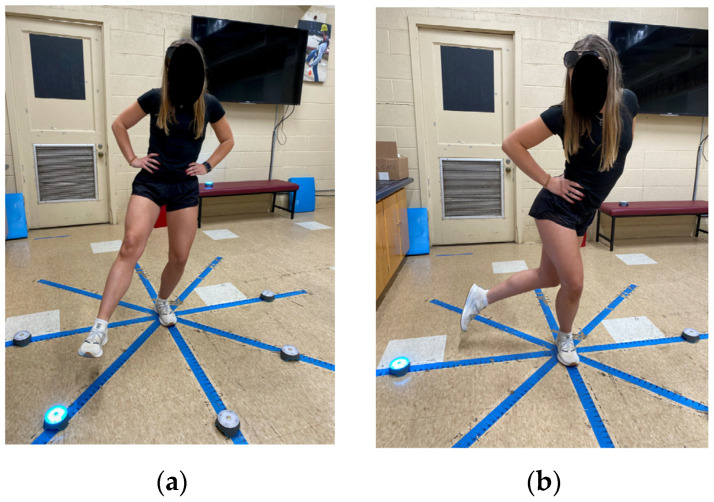
Participant performing SEBT with the left leg stance condition (**a**) and the right leg stance condition (**b**), while simultaneously activating the BlazePod during the mSEBT cognitive-motor task.

To evaluate balance ability in a cognitive/motor task, each participant performed the SEBT, which consisted of standing on one leg [Left Leg Reach (LLR) or Right Leg Reach (RLR)] and reaching as far as possible in 5 directions [Anterior, Anterior-Medial, Medial, Lateral, Anterior-Lateral], one by one, repeated for three trials for each leg. Reaching distance was recorded in inches based on the tape measure on the floor. Blazepod™ (BlazePod Inc., Miami, FL, USA) sensors were then placed on the average maximum reaching distances for each participant. Blazepod^TM^ sensors are reactive lights seen in Figure 1, which turn off or deactivate with contact. They are controlled via smart phone applications using Bluetooth low-energy (BLE) technology and allow for full customization of 10 colored lights which record time variables from activation, until deactivation. In this scenario, pods were activated automatically and randomly with a timer and deactivated with the impact of the foot touching the top part of the pod (see Figure 1). BlazePod™ technology has been widely used by strength conditioning professionals, and more recently, the test-retest reliability of a single leg striking the BlazePod™ task was tested and reported to have moderate to excellent levels of reliability [28].

The SEBT was performed again for three trials, but required participants to strike the Blazepod™ sensors as soon as they lit up (randomly turned on) as the mSEBT. Posterior, posterior-lateral and posterior-medial directions were not tested as participants were unable to see the sensors. All these measures were considered as pre-workload (PRE). The fatigue protocol had been used in a recent study [29] and consisted of three sets of three different lower extremity exercises: Ten unilateral bodyweight calf raises, 20 standard bodyweight squats, 20 standard bodyweight lunges. Immediately following this, the participants repeated all the testing procedures mentioned above as a post-workload measure (POST). RTs were averaged within each reach direction to form a mean RT measure for each trial as a measure of dynamic balance ability and cognitive motor learning.

### 2.3. Statistical Analyses

A 2 (fatigue state [PRE vs. POST]) × 2 (leg [RLR vs. LLR]) × 3 (Trial 1 vs. Trial 2 vs. Trial 3) repeated measures ANOVA was performed to test for differences in mean RT across fatigue states, leg, and trials. Significant interactions were decomposed with follow-up post hoc tests with a Holm correction. All statistical analyses were conducted in JASP (v0.15). An alpha level of *p* < 0.05 was considered statistically significant. Effect sizes were calculated as partial eta square (η_p_^2^) for repeated-measures ANOVAs and Cohen’s d for post hoc tests. We classified the magnitude of effects using the guidelines of the Refs. [30,31], respectively.

## 3. Results

**Figure 2:**
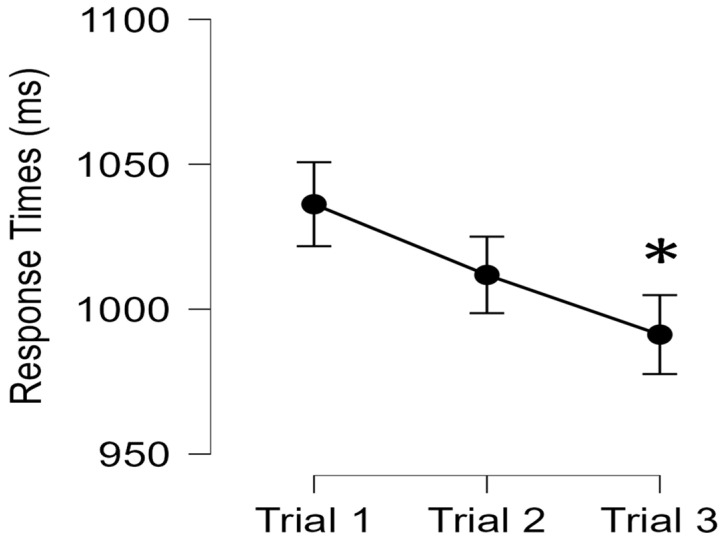
Mean response time across the three trials, pooled between fatigued and leg reach conditions. Represented as mean ± standard error. * represents a significant difference from Trial 1 (*p* < 0.05).

The results for the SEBT are presented in Figure 2 with descriptive statistics presented as mean ± standard deviation for RT (ms) in Table 1. There was a significant main effect for trials (*p* = 0.023, η_p_^2^ = 0.223), such that Trial 3 demonstrated significantly faster response times compared to Trial 1 collapsed across the leg and fatigue states (*p* = 0.021, d = 0.645), while no differences existed between Trials 1 and 2 (*p* = 0.077, d = 0.474) or Trials 2 and 3 (*p* = 0.202, d = 0.334). No other significant interactions or main effects existed between fatigue states or the leg (*p* > 0.05).

## 4. Discussion

The purpose of the study was to test the cognitive-motor ability of dynamic balance, by modifying the traditional SEBT to include a cognitive response time task as the mSEBT, in both the right and left lower extremities as well as before and after a low-intensity localized muscular fatiguing workload. Findings from the study revealed significantly faster RTs during the mSEBT with each successive trial. There were no significant differences as a consequence of the localized muscular fatiguing workload in either the right or left lower extremities. Finally, the study also suggests that a light response task can be added to the traditional SEBT to assess the cognitive-motor ability of a dynamic balance assessment, using the proposed mSEBT.

### 4.1. Response Time and Postural Control

The primary results showed a significant decrease in RT from Trials 1 to 3 with the mSEBT. The present study contributes unique information suggesting that practitioners utilizing the mSEBT to assess dynamic balance may consider utilizing at least 2–3 trials. The decrease in reaction time from Trials 1 to 3 in the BlazePod^TM^ mSEBT could have been from different underlying mechanisms. One likely causation is that there was a learning effect. Given that the traditional SEBT requires four trials prior to any legitimate data collection, this modified version would likely need equivalent pretests to provide reliable data [32]. However, participants had received nine total recorded trials of the SEBT—three to find the average distance for pod placement, and another three in both fatigue states conditions with the pods task present. A second theory is that the SEBT and our mSEBT with the RT element are considered completely independent of one another and would require additional practice in order to acclimate participants to this new general motor program. However, if this is not the case, the decrease in reaction time between Trials 1 and 3 could represent training improvements from this cognitive motor dual-task itself, which, if done repetitively, can show improvements in both postural control and RT as both a single and dual task [23].

A third proposed theory for the decreased RT found in this study is a change in motor control strategies between the three trials. This concept has been previously addressed by van Dieën et al. [33], who demonstrated a correlation between changes in performance between pre-training trials and the first post-training trials on an unstable surface, indicating improvements in center of mass excursions. Results showed a significant correlation between the pre-training COM excursion and visual manipulation [33]. These findings suggest that when vision is not altered or is increased, improvements in acute balance performance can occur due to an increased reliance on the visual system. Therefore, it can be interpreted that when there is a decrease in time spent correcting faulty balance and visual feedback is unattenuated, RT can decrease.

It is worth noting that the present participants were young, healthy adults with no previous or recent musculoskeletal injury. Thus, the SEBT protocol may have been a simplistic skill, which is to suggest a very acute, immediate learning effect seen in reductions in the reaction time from Trials 1 to 3. This may be further understood by examining attentional demands as they relate to learning. For instance, Kahneman [34] proposed that the simpler the demands of a given task, the greater the performance, even if the task is new. This is further supported by Gabbett et al. [11] who found that more skilled rugby players had lower attentional demands during fundamental rugby drills than less skilled players. It has also been seen during a dual-task cognitive motor interference experiment with balance and reaction time activities that the direction of attentional focus between cognitive and motor tasks significantly impacts how participants perform both single tasks individually. Focusing on the reaction time task in this study increased both reaction time and postural control variables [23]. Thus, the combination of increased reliance on the visual system and the nature of healthy adults performing what may be perceived as a simple task may account for the acute ability of RT to be improved, independent of fatigue. Nevertheless, as this hypothesis is merely a speculation, it requires future work.

### 4.2. Localized Muscular Fatigue

Interestingly, acute localized muscular fatigue due to the performed physical workload did not negatively impact RT, which suggests that the intensity of the workload may not be sufficient to cause a reduction or an impairment of the sensory and motor responses [26]. Muscular fatigue occurs as a result of any failure in the process of a muscular contraction which leads to reduced capabilities to produce adequate force [3,35]. Researchers suggest that fatigue, specifically at the ankle, causes a decrease in proprioception and balance control due to a decrease in the sense of position [2,35]. This disruption in joint awareness is attributed to an increased threshold of muscle spindle discharges caused by fatigue and a decrease in action potential velocities along the sensory pathway [2,26]. Voluntary movement is therefore affected as fatigue attenuates the motor intention at the central level before it is transferred to the working muscles via the motor pathways [36], and as a result, researchers have theorized that fatigue causes a discrepancy between the predicted motor plan and the reafferent feedback due to the deficient motor command [36]. Interestingly, the results of the present study demonstrated that fatigue had no effect on RT, which appears contradictory to previous studies [37] but may be purely due to the lower intensity nature of the workload used and the young healthy population tested in the current study.

### 4.3. Protocol

This novel integration of a cognitive RT task to the traditional balance test was performed with the intent of providing various health care professionals with a more holistic method of assessing postural control, reaction time, and dual-task ability of participants using the proposed mSEBT. The mechanisms of the mSEBT presented here strongly resemble that of the limits of stability protocol on the NeuroCom and Biodex, in which one displaces their center of pressure to eight predetermined targets at 45-degree increments over 360 degrees [38] as quickly as possible. Essentially, the mSEBT is the non-equipment-based equivalent of these dynamic balance force plate assessment protocols, but only with an addition of a light response task to make it more of a cognitive-motor assessment of dynamic balance. This addition to the traditional limits of stability protocols provides a cognitive task in addition to the pre-existing motor task creating a DT paradigm that could be used in training to elicit improvements beyond that of either single tasks in isolation [13,20,21]. The NeuroCom and Biodex systems are much more advanced with regard to the variables collected, especially using center-of-pressure excursions from the force platforms. However, these instrumented computerized dynamic posturographies also come with high costs. The current version of the mSEBT offers a low-cost option for adding a cognitive-motor response task, that can be beneficial to all clinicians and health care professionals for the purposes of diagnosis, rehabilitation and prognosis in postural control and dynamic balance-based impairments.

### 4.4. Limitations and Future Directions

This study has several limitations, but also provides several opportunities for future research. First, the findings from the study needs to be interpreted with caution. Even though there was a significantly decreased response time from Trials 1 to 3, the acclimation period for this new protocol must be appropriately examined, especially with a greater number of trials performed in sequence and its impact on learning effects. Evaluation of how many trials it would take for participants to plateau in performance is warranted. With such a perspective, the mSEBT can be used as an assessment tool and as a balance training intervention. The current study also only tested healthy young adults with no serious history of musculoskeletal or neuromuscular abnormalities. The current study only recruited young, recreationally trained, and otherwise healthy individuals, as this was a novel modification of the traditional SEBT, commonly conducted among young athletic populations. However, testing for different age groups, different clinical conditions, such as musculoskeletal or neuromuscular abnormalities, different sporting populations, and so forth can provide greater insights into the use and benefits of the mSEBT.

The current study did not have any objective quantifiable measures of fatigue using physiological or biomechanical measures, or even subjective measures of fatigue and exertion. Future studies should incorporate both objective and subjective measures of fatigue to have a better understanding of the impact of physical workload and fatigue on the mSEBT. Finally, the current study did not incorporate other biomechanical measures along with the mSEBT. In the mSEBT, the dynamic balance reaching distance and response time variables were both measured based on the volitional control of our bodies center of mass about our base of support. Therefore, the addition of kinematic measures such as three-dimensional motion capture, kinetic measures such as using a force platform for the stance extremity, and using electromyography (EMG) for both stance and reaching lower extremity can provide a holistic approach to a dynamic balance assessment.

## 5. Conclusions

The current study attempted to incorporate a light response task to the traditional SEBT with the intent of making the SEBT a more complete tool in analyzing dynamic balance using a cognitive-motor approach, such as the mSEBT. Integration of response time and dual task ability is a critical element of postural control, involved in athletic competition and during ADL. Findings from the current study demonstrated that response times could be assessed in the mSEBT and that they were significantly faster with repeated trials, suggesting that the initial learning effects should be controlled by allowing performance to plateau prior to testing. No significant differences existed due to the physical workload or between the left and right lower extremities. The findings of the current study add a new element to dynamic balance testing that could be utilized as a clinical maker to facilitate diagnosis, training, rehabilitation, and prognosis. The mSEBT demonstrated a promising protocol for integrating response time and dual-task cognitive-motor interference into the traditional SEBT balance assessment, which are critical for the assessment and improvement of dynamic balance, and subsequently for identifying fall risks.

## Figures and Tables

**Table 1:** Descriptive statistics for response times.

Trials	Mean	Std. Dev
Trial 1	1036.20	100.65
Trial 2	1011.81	118.53
Trial 3	991.21	101.64

Response times (RT) in milliseconds (ms) presented as mean ± standard deviation (std. dev).

## Data Availability

Not applicable.
